# Nanocrystalline Cellulose Supported MnO_2_ Composite Materials for High-Performance Lithium-Ion Batteries

**DOI:** 10.3390/ma14216619

**Published:** 2021-11-03

**Authors:** Quang Nhat Tran, Thuan Ngoc Vo, Il Tae Kim, Ji Hyeon Kim, Dal Ho Lee, Sang Joon Park

**Affiliations:** 1 Department of Chemical and Biological Engineering, Gachon University, Seongnam 13120, Gyeonggi-do, Korea; tran.nhat147@gachon.ac.kr (Q.N.T.); vnthuanbk@gmail.com (T.N.V.); itkim@gachon.ac.kr (I.T.K.); jihyeon@gachon.ac.kr (J.H.K.); 2 Department of Electronics Engineering, Gachon University, Seongnam 13120, Gyeonggi-do, Korea; dhlee@gachon.ac.kr

**Keywords:** manganese dioxide, nanocrystalline cellulose, lithium-ion batteries, nanocomposite, discharge capacity

## Abstract

The rate capability and poor cycling stability of lithium-ion batteries (LIBs) are predominantly caused by the large volume expansion upon cycling and poor electrical conductivity of manganese dioxide (MnO_2_), which also exhibits the highest theoretical capacity among manganese oxides. In this study, a nanocomposite of nanosized MnO_2_ and pyrolyzed nanocrystalline cellulose (CNC) was prepared with high electrical conductivity to enhance the electrochemical performance of LIBs. The nanocomposite electrode showed an initial discharge capacity of 1302 mAh g^−1^ at 100 mA g^−1^ and exhibited a high discharge capacity of 305 mAh g^−1^ after 1000 cycles. Moreover, the MnO_2_-CNC nanocomposite delivered a good rate capability of up to 10 A g^−1^ and accommodated the large volume change upon repeated cycling tests.

## 1. Introduction

Recently, rechargeable lithium-ion batteries (LIBs) with metal oxide-based materials, such as iron oxide, tin dioxide, cobalt oxide, titanium dioxide, manganese dioxide, and nickel oxide, have attracted significant interest as anodes. LIBs owing metal oxide anode materials are promising due to their favorable chemical properties and high performance [1,2,3,4,5,6,7,8,9,10]. A wide range of transition metal oxides, sulfides, selenides, fluorides, nitrides, and phosphides have been approached as conversion-type anode materials (CTAMs) for LIBs, which greatly increase the materials for high performance LIBs. Furthermore, many CTAMs are found in their natural forms and have low production costs in comparison with alloying-type anode materials. In addition, compared to graphite anodes with a low Li-intercalation potential, CTAMs show better safety of LIBs by avoiding the problem of lithium dendrite formation. Moreover, silicon-based and phosphorous (P) materials coated with magnesium (Mg) may be considered to be promising for Li metal as an anode and cathode for the large market of next generation LIBs, and could be a replacement for graphite and graphitic carbon as electrode materials [11,12,13,14,15]. One of the transition metal oxides and conversion-type anode materials, manganese dioxide (MnO_2_), is not only the most stable form of MnO_x_ compounds but also shows the highest theoretical capacity of 1232 mAh g^−1^ among manganese oxides. MnO_2_ is particularly attractive as an electrode material in LIBs because of its high energy density, small voltage hysteresis, high abundance, low conversion potential, and environmental friendliness [9,16,17,18,19,20,21,22,23]. However, drawbacks, such as poor electrical conductivity and large volume expansion during charge–discharge cycling, limit the performance of LIBs. The poor electrical conductivity causes poor rate capability, and the large volume expansion during cycling significantly affects the battery stability [16,24]. This limitation results in a significant drop in the capacity at high current densities. Many approaches have been proposed, such as the synthesis of nanostructured MnO_2_, to overcome these problems and to achieve improved battery performance [16,17,25,26,27].

The size and the morphology of nanostructured MnO_2_ particles are beneficial for improving the contact area between the electrode and the electrolyte, to prevent structural distortion caused by Li^+^ insertion/extraction, and to reduce the diffusion pathways of Li ions. However, the high surface energy of metal nanoparticles leading to self-aggregation could decrease the productive contact area between the conductive additives, electrolyte, and active materials [28]. Moreover, side reactions between the active materials (owing to their high surface area) and the electrolyte shorten the cycle life of the battery and raise safety concerns [28]. Forming a nanocomposite with electrically conductive carbon additives is a valid method to overcome the poor electrical conductivity of MnO_2_ [9,25,26,29,30,31,32,33,34,35,36,37]. Among the carbon-based materials, pyrolyzed nanocrystalline cellulose (CNC) not only maintains the structural stability of anode materials, but also enhances the electrical conductivity of MnO_2,_ owing to its excellent colloidal stability [37,38]. Furthermore, CNC is uniformly dispersed in solution owing to the presence of negative surface charges; this prevents the aggregation of metal oxide nanoparticles during the synthesis process [39]. The low cost, light weight, and favorable physicochemical robustness of CNCs are expected to enable their wide commercial applications.

In this work, we prepared MnO_2_-CNC nanocomposite with porous nanosized MnO_2_ to exploit the low external surface area and the low surface energy of nanostructured particles, and could prevent side reactions and self-aggregation. Pyrolyzed CNC enhances the electrical conductivity of the anode nanocomposite and also alleviates stress due to volume changes to improve the material electrochemical performance during the cyclability. Moreover, pyrolyzed carbon material effectively prevents nanoparticle aggregation and acts as a dispersant for the synthesis of nanosized MnO_2_. Thus, the MnO_2_-CNC nanocomposite is expected to be a promising electrode material to provide improved capacity and the stable cycle performance of LIBs. 

## 2. Materials and Methods

### 2.1. Materials

The CNC suspension obtained from SK Innovation Co. Ltd. (Daejeon, Korea) was pyrolyzed at 800 °C (as described in our previous paper [40]) and was used as a source of CNC for synthesizing the nanocomposite from MnO_2_ and CNC. Manganese (II) sulfate hydrate (MnSO_4_·xH_2_O) and potassium permanganate (KMnO_4_), purchased from Sigma–Aldrich Co. Ltd. (St. Louis, MO, USA), were used in this study. 

### 2.2. Synthesis of MnO_2_-CNC Nanocomposite

The MnO_2_-CNC nanocomposite was prepared by a modified approach, as previously described [31]. Specifically, 0.2 g of pyrolyzed CNCs was dispersed in 35 mL (10 mM) of MnSO_4_ by ultrasonication for 10 min. The obtained suspension was maintained at a controlled temperature of 80 °C, and was magnetically stirred for 20 min. Then, 150 mL of aqueous solution of (33 mM) KMnO_4_ was heated at 80 °C and gradually added to the previous mixture. The resulting suspension was stirred under a controlled temperature of 80 °C for 15 min. The obtained sample was washed, filtered, and dried at 100 °C for 10 h.

### 2.3. Materials Characterization

The nanocomposite materials were characterized using an X-ray diffractometer (XRD) (Rigaku/Smartlab, Tokyo, Japan) operated at 40 kV and 30 mA with a Kβ filter for Cu. The XRD profile was examined with a scan rate of 5.0° min^−1^ from 10 to 80° for 2θ angle. The structural and morphological characterizations of the samples were examined by using scanning electron microscopy (SEM; S-4700, Hitachi Ltd., Tokyo, Japan) and transmission electron microscopy (TEM; Tecnai, F30S-Twin, Hillsboro, OR, USA), and elemental maps were obtained by energy dispersive X-ray analysis (EDX). Thermogravimetric analysis (TGA) was applied for analyzing the composition of the nanocomposite with a temperature increase rate of 10 °C min^−1^ under atmospheric condition. Brunauer –Emmett–Teller (BET) specific surface areas of MnO_2_-CNC composites were determined by N2 adsorption at 77.3K (Micromeritics, ASAP 2020).

### 2.4. Electrochemical Performance Measurement 

**Figure 1:**
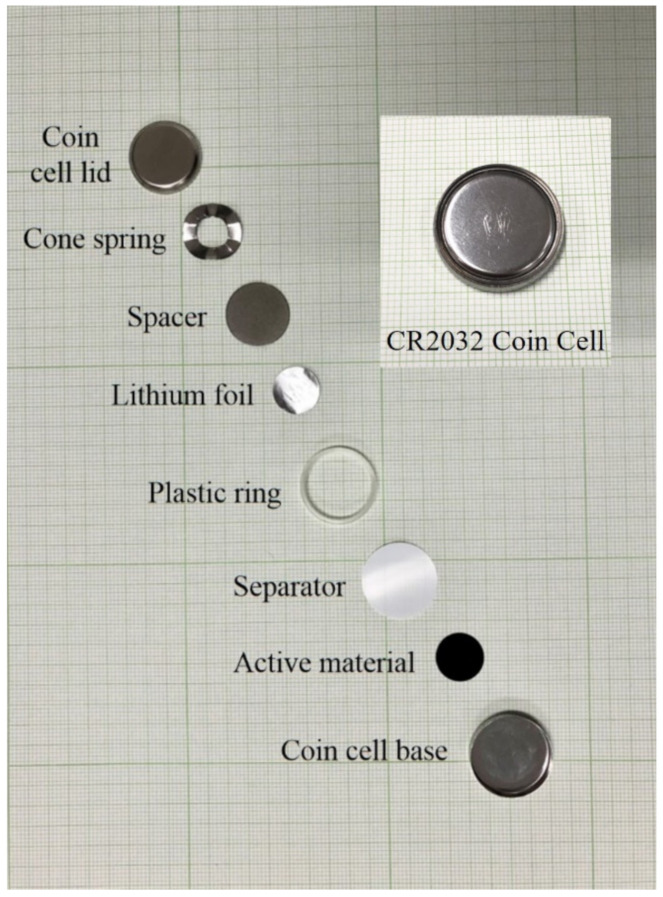
The component of the testing LIB coin cell.

To produce the working electrode (mass loading of 0.088 mg/cm^2^), a slurry containing 70 wt% of the nanocomposite as active materials, 15 wt% Poly(vinylidene fluoride) (PVDF), 15 wt% Super P, and N-methyl pyrrolidone, was prepared and coated on a Cu foil (r = 0.6 cm). The electrodes were maintained at 60 °C for 3 h and then dried overnight at 70 °C in a vacuum oven. The electrochemical performance of the obtained MnO_2_-CNC nanocomposite was directly evaluated using coin cells (CR2032) fabricated in a glove box filled with argon. The cells consisted of a coin cell base, a polyethylene membrane as the separator, a plastic ring, a lithium foil as the counter electrode, a spacer, a coin spring for improving contact and the coin cell lid. A mixture of ethylene carbonate/diethylene carbonate (1:1 in volume) dissolved with 1 M LiPF_6_ was used as the electrolyte solution (see Figure 1). Cyclic voltammetry (CV) were tested from 0.01 to 3.0 V at a scan rate of 0.1 mV s^−^^1^. The battery cycler (WBCS3000, WonAtech) system was used to examine the cell performance experiments and cycle performance tests under a constant current density of 100 mA g^−1^ at 0.01–3.0 V (vs. Li/Li^+^) at room temperature. Subsequently, the rate performance tests were performed using various current densities in the range 100–10,000 mA g^−1^.

## 3. Results and Discussion

**Figure 2:**
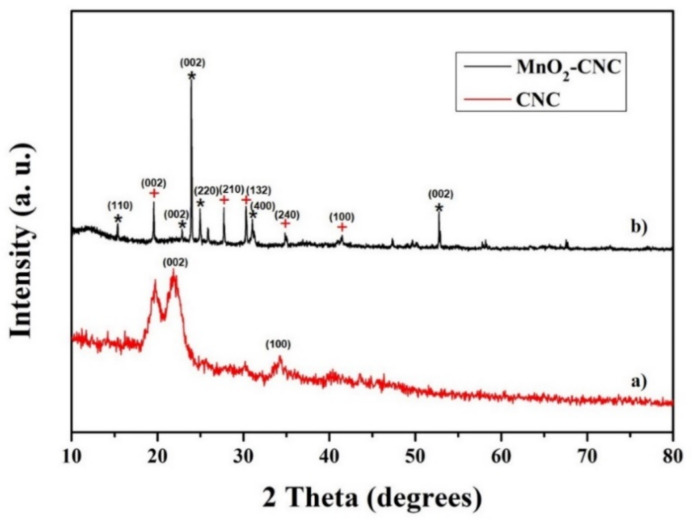
Powder XRD characteristic of (**a**) CNC and (**b**) MnO_2_-CNC nanocomposite.

Figure 2a shows the XRD pattern of CNC. The obtained peaks are centered at 19.8, 22.6, 34, and 40.2°, which match well with the data provided by SK Innovation Co. Ltd. The presence of sharp peaks designates the crystalline nature of CNC. The XRD profile of MnO_2_-CNC nanocomposite in Figure 2b shows the characteristic peaks of native cellulose and cellulose at 19.5, 27.7, 31, 34.8, and 41.4°, which totally agrees with the ICSD data (PDF 03-0289 and 03-0226). The peaks centered at approximately 15.8, 22.8, 25, 32, 36.9, and 52.7° correspond to the (110), (002), (002), (220), (400), and (002) planes, respectively, of ɛ-MnO_2_ (ICSD PDF 12-0141). The peaks of MnO_2_ are sharper and more intense than those of CNC, indicating the formation of crystal phases ɛ-MnO_2_ and the coexistence of nanocrystalline phases (CNC) in the composite [30]. This is beneficial for increasing the electrical performance of the metal oxide electrode of the battery. The nanocrystalline phase is also expected to prevent the combination of nanosized MnO_2_ particles and to improve the contact between the electrode materials and the electrolyte, emerging in favorable cycle performance.

**Figure 3:**
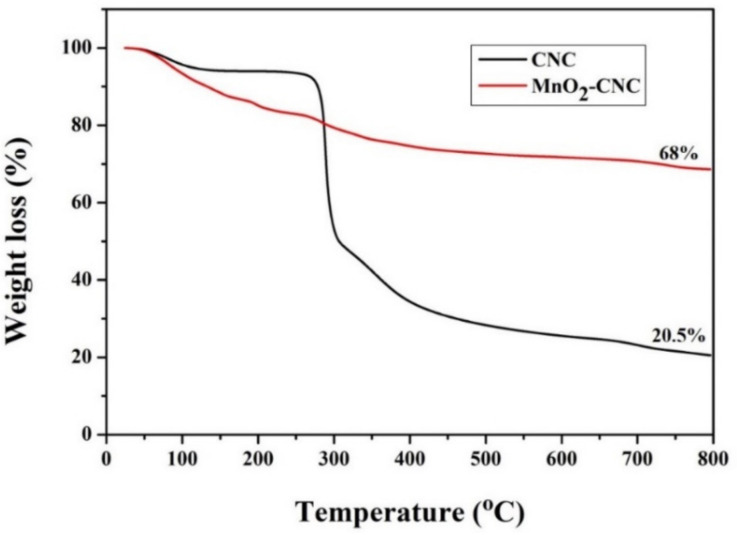
TGA curves of CNC and MnO_2_-CNC composite.

TGA was used to examine the chemical composition of the MnO_2_-CNC nanocomposite. The TGA patterns of CNC and MnO_2_-CNC nanocomposite are shown in Figure 3. The TGA curve of CNC shows a mass loss of approximately 7% below 250 °C, corresponding to impurities and adsorbed water present in the air. The pyrolysis of CNC is recognized by the major weight loss between 250 and 750 °C [40]. The extant material weight is 20.5%, which is considered to be that of pyrolyzed CNC. The TGA curve of the MnO_2_-CNC composite shows a weight loss of 17% below 250 °C, corresponding to adsorbed water, trace amounts of oxygen, and easily oxidizable matter present in the air. The weight loss gradually decreases from 250 to 750 °C, which is believed to be due to the burning of CNC in air [41]. A residual weight of 68% is obtained for the MnO_2_-CNC composite. Therefore, the mass percentage of residual MnO_2_ is calculated to be 47.5%.

**Figure 4:**
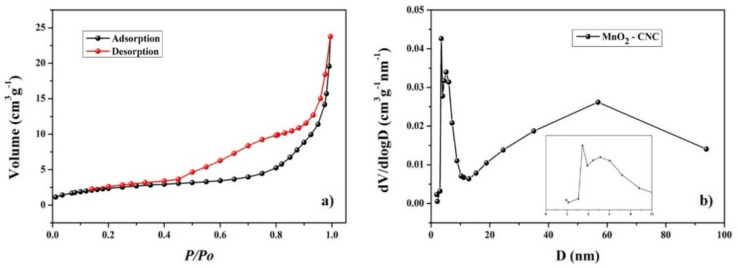
(**a**) Nitrogen adsorption-desorption isotherm and (**b**) BJH pore size distribution of the composite.

The porous surface property of composite was determined by N_2_ isothermal adsorption and desorption, and the obtained results are shown in Figure 4. The pattern shows a typical IV isotherm curve with hysteresis loops, indicating the mesoporous structure of the composite [42]. The BJH adsorption and desorption average pore diameter are 17.56 and 10.85 nm, respectively. A wide hierarchical pore size distribution centered at 3.4 nm, 5.1 nm, and 56.9 nm could offer more approachable active sites for the intercalation of Li ion and electrochemical reaction to enhance the specific capacity of the electrode. On the other hand, the uniformly dispersed MnO_2_ on the CNC can effectively reduce the aggregation of MnO_2_ nanoparticles and also the mesoporous structure can prevent the volume expansion of MnO_2_, which improves the cycling stability of the electrode [43].

**Figure 5:**
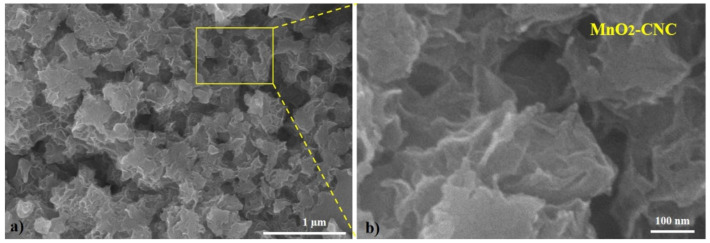
(**a**) Typical and (**b**) high-magnification SEM images of the MnO_2_-CNC nanocomposite.

The SEM system was used to study the surface morphology of the MnO_2_-CNC nanocomposite, and the images are shown in Figure 5. The composite exhibits a uniform hierarchical structure consisting of MnO_2_ nanoparticles (Figure 5a). The hierarchical morphology of nanocomposite was recorded by high-magnification SEM image (Figure 5b), clearly confirming that the surface of the nanocomposite is porous. This indicates that MnO_2_ is covered by CNC, which prevents aggregation of nano-sized MnO_2_ and improves the electrode performance.

**Figure 6:**
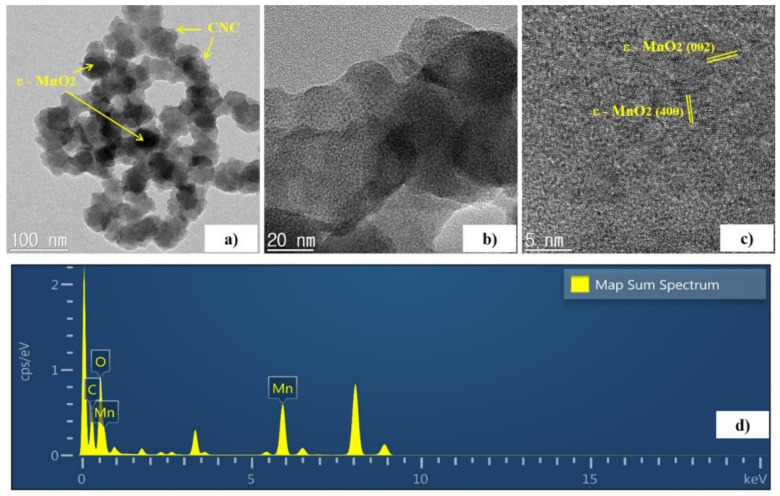
(**a**) Typical, (**b**,**c**) high-magnification TEM images and (**d**) EDX pattern of MnO_2_-CNC nanocomposite.

**Figure 7:**
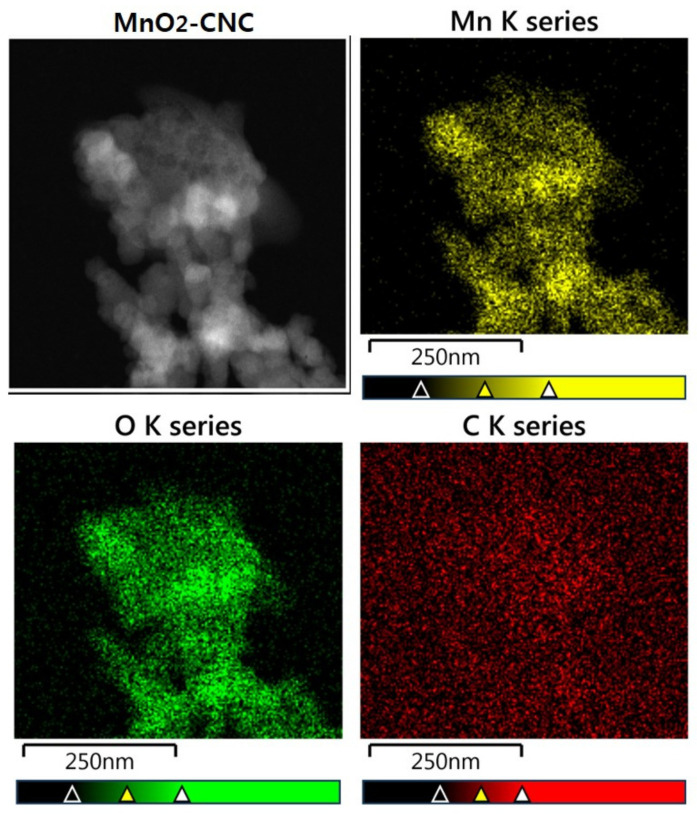
TEM elemental mapping of the MnO_2_-CNC nanocomposite.

The more detailed morphology and nanostructure of the MnO_2_-CNC nanocomposite were investigated by TEM and high-resolution TEM (HRTEM). Figure 6a,b indicate the obtained MnO_2_-CNC nanocomposite exhibits uniformly distributed MnO_2_ nanoparticles covered with CNC, which is in accordance with the SEM results. The MnO_2_ nanoparticles exhibited an average particle size of 20–25 nm with a flower-shape. Moreover, the nanocomposite is efficiently segregated from the supporting CNC layer. The crystalline structure of MnO_2_ is evident from Figure 6c. The (400) and (002) planes of MnO_2_ are consolidated by lattice fringe spacings of 0.242 and 0.495 nm, respectively, which consistently agrees with the XRD results. The HRTEM images also showed the presence of a macroporous layer of the carbon substrate surrounding the MnO_2_ nanoparticles. Hence, the combination of the crystalline structure of nano-sized ɛ-MnO_2_ and the porous surface modification by CNC in the MnO_2_-CNC nanocomposite is expected to extend and improve the cycling stability, rate performance, and initial reversible capacity of the nanocomposite, used as electrode materials in LIBs. Furthermore, the presence of MnO_2_ particles and CNC in the nanocomposite is confirmed by the peaks from the EDX spectrum (Figure 6d), which corresponds to Mn, O, and C. The TEM elemental mapping of the MnO_2_-CNC nanocomposite, as shown in Figure 7, further confirmed the uniform distribution of MnO_2_ and CNC. 

**Figure 8:**
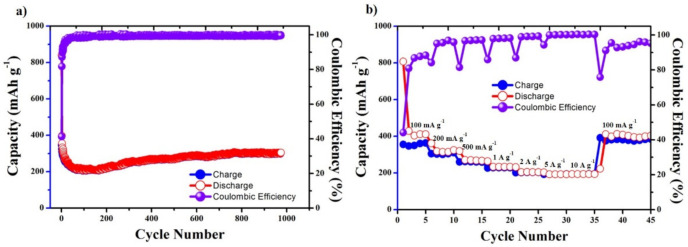
(**a**) Cyclic performances of the MnO_2_-CNC nanocomposite electrodes at 100 mA g^−1^ and (**b**) Rate performance of MnO_2_-CNC nanocomposite electrodes at various current densities ranging from 0.01 V to 3 V.

A potential range 0.01–3.00 V was applied in LIBs to investigate the electrochemical properties of the MnO_2_-CNC nanocomposite, performed as an anode material. The discharge and charge capacities and the long-term cycle performance test of the MnO_2_-CNC nanocomposite electrode are shown in Figure 8a. The experiment tests were carried out at 100 mA g^−1^ for 1000 cycles. Except for a significant decrease in the capacity during the first five cycles and a slight reduction in the stability over the next 100 cycles, the capacity increases to a steady value of approximately 300 mAh g^−1^ in the subsequent thousand cycles. The nanocomposite exhibits an enhancement capacity of 30% compared to the obtained result at the 100th cycle with a good capacity of 305 mAh g^−1^ after 1000 cycles. In addition, the charge–discharge capacity of the nanocomposite tends to a gradual increase in the cycle after decreasing in the first cycles; this is a characteristic phenomenon for metal-oxide nanocomposite anodes [9,44,45,46,47,48]. The pulverization of the nanocomposite during the lithiation process decreases the typical initial capacity, and the metal oxides’ interfacial storage mechanism, resulting from the activation process on cyclability causing an increase in the capacity. Despite the loss of crystallinity of the nanosized MnO_2_ particles during the cyclability process of anode nanocomposites, the incorporated CNC was more tolerant and flexible than the metal oxide structure, which helped the nanocomposite material anode to easily accommodate volume changes during lithiation and increase the capacity after cycling [34,35,36,37,38,39]. Thus, the nanocomposite electrode material exhibited a good stable property on cyclability, delivered a reversible capacity of 305 mAh g^−1^ after 1000 cycles, and retained 36% of its inception capacity with a Coulombic efficiency of over 99.0%, which establishes a significant stable effective impact on the cyclability of nanocomposite. These results again confirm the supporting role of CNC, which minimizes the volume expansion to prevent the collapse of the MnO_2_ structure, as a result of charging and discharging over many cycles.

A different current density range of 0.1, 0.2, 0.5, 1, 2, 5, and 10 A g^−1^ was applied to examine the rate performance and charge and discharge capacity of the MnO_2_-CNC nanocomposite. Figure 8b shows the obtained results from the test. The nanocomposite electrode owns average reversible capacities of 492, 324, 279, 239, 210, 195, and 193 mAh g^−1^, respectively, for five cycles. Following the rate capability test, the specific applied current of 100 mA g^−1^ is maintained for the next 10 cycles, and the electrode average capacity within 10 cycles increases to a value of 403 mAh g^−1^, indicating the stability and good rate-cycling performance of the electrode at various current densities. 

**Figure 9:**
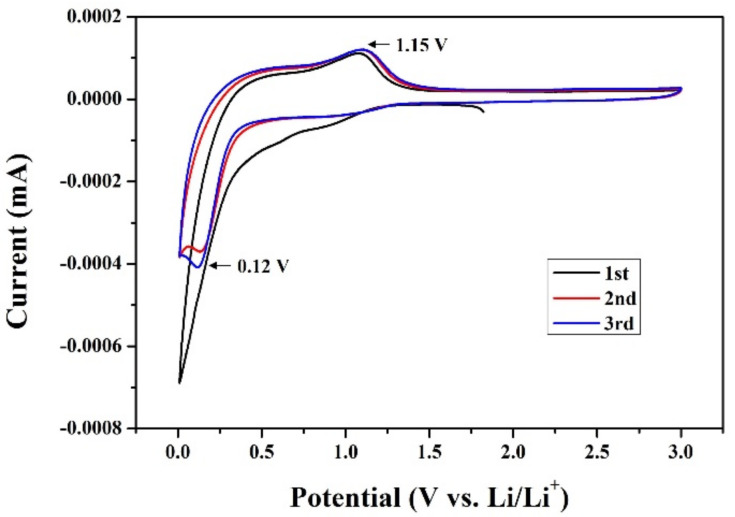
The Cyclic voltammetry (CV) curves of MnO_2_-CNC nanocomposite electrodes.

The CV curves of three initial cycles from 0.01 to 3.0 V range at a scan rate of 0.1 mV s^−1^ are shown in Figure 9. It can be clearly observed that the first CV curve shape and composite area differ from those of the next two cycles. On the first cathodic cycle, there is no peak that can be observed; however, in the following anodic process, there appears a significant peak at 1.15 V coresponding to the oxidation of MnO_2_ nanoparticles during the delithiation reactions. From the second cycle, a reduction peak at 0.12 V can be confirmed, indicating that the carbon-based materials may form a stable SEI film [37,38,39,40]. Moreover, a boarder oxidation peak can still be observed at 1.15 V from the second cycle and is almost overlapped, which suggests that MnO_2_-CNC has a good stability in structure and electrochemial property after the first cycle.

**Figure 10:**
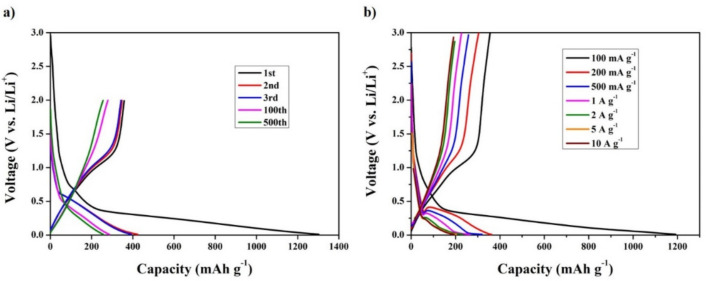
The charge–discharge profiles of the MnO_2_-CNC nanocomposite at (**a**) 100 mA g^−1^ and at (**b**) various current densities.

Figure 10a,b respectively show the typical charge–discharge capacities of the obtained nanocomposite electrode during cycling at a current density of 100 mA g^−1^ and at different current densities in the potential range 0.1–3.0 V (Li/Li^+^). The initial charge capacity and discharge capacity of the nanocomposite electrode are 1302 and 398 mAh g^−1^, respectively. The corresponding initial Coulombic efficiency is 30%. However, these capacity values are superior to the theoretical capacities of MnO_2_ and CNC. This phenomenon can be explained by the increase in the irreversible capacity of the electrode, caused by the decomposition of the electrolyte during the first discharge process and the formation of a solid–electrolyte interface (SEI) layer on the surface. Moreover, the supporting role of pyrolyzed CNC in the nanocomposite also contributes to the superior initial capacities [37,40]. The obvious discharge platform observed at approximately 0.7 V during the first discharge process, which can be recognized from Figure 10a, is attributed to the decomposition of the electrolyte and the formation of the SEI film. In the subsequent cycles, the platform at 0.7 V disappears, demonstrating that the SEI film is stably formed after the first cycle. In addition, the electrodes’ irreversible capacity decreases significantly during the first cycle, which is a typical phenomenon in LIBs based on metal oxides. Furthermore, the charge and discharge profiles of the 2nd, 3rd, 100th, and 500th cycles show similar shaped curves. The trend in the reversible capacity of the nanocomposite after 500 cycles is similar to that after the 100th cycle, indicating that the nanocomposite material exhibits good electrochemical stability and stable operation cyclability from the second cycle.

Figure 10b shows the initial discharge–charge profiles of nanocomposite electrode at different current rates. The recorded capacities are 1191, 361, 319, 263, 231, 204, and 193 mAh g^−1^ at 0.1, 0.2, 0.5, 1, 2, 5, and 10 A g^−1^ current densities, respectively. At low current densities, the plateaus around 0.4 V during the discharge process and at approximately 1.25 V during the charging process are observed, which are similar to those observed in Figure 10a for the cycling test. At higher current rates, the plateaus in the charging process disappear, but the plateau in the discharge process is maintained, which evidences that electrochemical redox reactions mainly influence the lithium storage process at high current densities. However, the shapes of the discharge–charge pattern are representative of the conversion reactions of transition metal oxide-based electrodes, and the same shape is maintained at diverse current densities. This further demonstrates the favorable performance of the nanocomposite material at high current rates.

**Figure 11:**
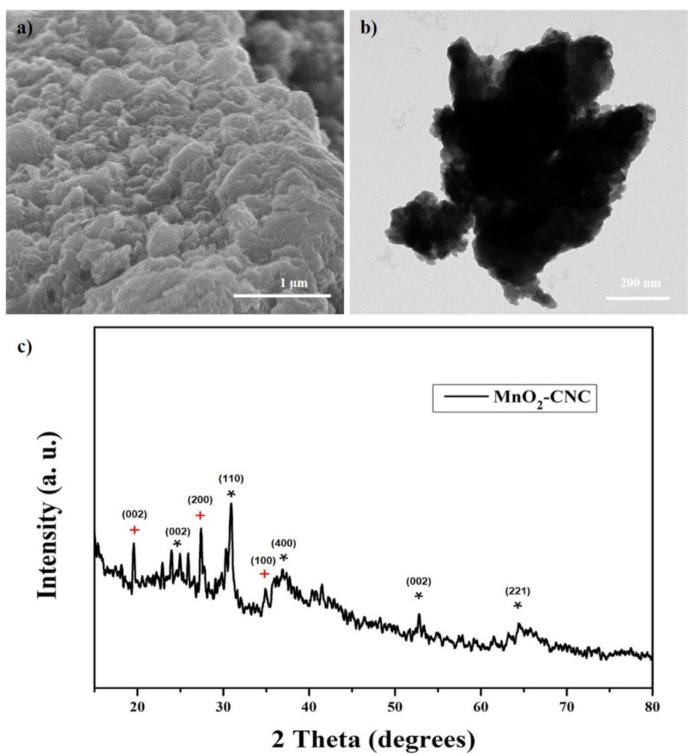
(**a**) SEM, (**b**) TEM and (**c**) XRD patterns of MnO_2_-CNN composite electrodes after 1000 cycles.

To further investigate the cycling influence on active material properties, SEM, TEM images, and XRD composite result after 1000 cycles were obtained and are shown in Figure 11. It can be observed from SEM and TEM images (Figure 11a,b) that the electrode could not maintain the good material structure and the aggregation appears after 1000 cycles. However, the porous structure and the carbon surrounding the MnO_2_ nanoparticles could be observed after 1000 cycling, which again confirms that CNC still covers and reduces the aggregation and volume expansion of MnO_2_. Moreover, the XRD result (Figure 11c) shows that broader and clear peaks correspond to the appearance of CNC and MnO_2_ nanoparticles.

In order to compare our research work with previous reports, which employ the nanocomposites of MnO_2_ and carbon-based materials working as the LIB anode, the electrochemical performances of LIBs are summarized in Table 1. Several electrically conductive carbon materials, such as carbon nanotube, graphene, and carbon nanohorns, have been used as substrate materials to improve the poor electrical conductivity of MnO_2_ and the anode structure stability. The results show that the new nanocomposite made of electrically conductive carbon materials and MnO_2_ can deliver a high initial capacity, with some reports exhibiting a better capacity than that of our work for the first 20~50 cycles. However, many of these materials show a significant drop in capacity in following cycles and still suffer from a stable cycle performance. On the other hand, our fabricated nanocomposite not only provides a good initial capacity, but also improves the material electrochemical performance during the extended number of cycles. In addition, the CNC effectively protects the structural stability of the anode material, leading to a better cycle stability.

## 4. Conclusions

In summary, we proposed a successful synthesis of a nanocomposite material of nanosized MnO_2_ supported by pyrolyzed nanocrystalline cellulose (CNC). The obtained nanocomposite-based anode exhibited an excellent cycling stability, rate performance, and capacity for LIBs. CNC not only improved the nanostructure by preventing the aggregation of MnO_2_, but also effectively assisted the large volume expansion upon cycling. The pyrolyzed CNC clearly contributed to an enhancement of the electrode performance, especially at high current densities. The obtained electrode shows an average reversible capacity of 193 mAh g^−1^ at 10 A g^−1^ current. The composite materials exhibited a stably increasing discharge capacity of 305 mAh g^−1^ after 1000 cycles and retained 36% of its inception capacity with a Coulombic efficiency of over 99.0% at 100 mA g^−1^. Moreover, the presence of pyrolyzed CNC is confirmed to overcome the drawbacks of nanosized MnO_2_ and maintains its structural stability, leading to a better capacity at high current densities, and the stable and excellent long-term cycling performance of nanocomposite as the electrode material of LIBs. Thus, the MnO_2_-CNC nanocomposite is an efficient electrode material for improving the electrochemical performance of next-generation LIBs.

## Figures and Tables

**Table 1:** Summary of previous works for electrochemical performance employing MnO_2/_electrically conductive carbon material composite.

No.	Composite	Discharge Capacity	Performance Cycle	Current Density	Reference
1.	MnO_2_-CNC	305 mAh g^−1^	1000	100 mA g^−1^	Our work
2.	MOCNF (MnO_2_ coated carbon nanofiber)	545 mAh g^−1^	1000	1000 mA g^−1^	[9]
3.	MnO_2_ (LMO)/PEDOT/Graphene	948 mAh g^−1^	15	50 mA g^−1^	[25]
4.	Graphene-MnO_2_-GNRs (Graphene nanoribbons)	890 mAh g^−1^	180	100 mA g^−1^	[26]
5.	MnO_2_/CNT hybrid coaxial nanotube	~500 mAh g^−1^	15	50 mA g^−1^	[30]
6.	MnO_2_/PGC (Porous graphic carbon)	692 mAh g^−1^	400	50 mA g^−1^	[31]
7.	MnO_2_/CNH (Carbon nanohorns)	565 mAh g^−1^	60	100 mA g^−1^	[32]
8.	MnO_2_/CNTs (Carbon Nanotube)	545 mAh g^−1^	1500	240 mA g^−1^	[33]
9.	Graphene/MnO_2_	225 mAh g^−1^	200	50 mA g^−1^	[34]
10.	MnO_2_/CNTs (Carbon Nanotube)	858 mAh g^−1^	260	200 mA g^−1^	[49]
11.	MnO_2_/GDY (Graphdiyne)	660 mAh g^−1^	120	200 mA g^−1^	[50]
12.	MnO_2_@HCN (Hollow carbon nanosphere)	604 mAh g^−1^	200	60 mA g^−1^	[51]
